# Hypercalcemia of Malignancy: An Atypical Presentation of Endometrial Carcinoma

**DOI:** 10.7759/cureus.70115

**Published:** 2024-09-24

**Authors:** Sarah J Choi, Petchpailin Sittirat, Noelle G Cloven, Shovendra Gautam

**Affiliations:** 1 Graduate Medical Education, Baylor Scott and White All Saints Medical Center, Fort Worth, USA

**Keywords:** endometrial carcinoma, hypercalcemia, hypercalcemia of malignancy, malignancy, malignancy-related hypercalcemia

## Abstract

Hypercalcemia of malignancy associated with lung, gastrointestinal, and hematologic malignancies is well-described in the literature but has rarely been reported with gynecologic cancers. Even among gynecologic malignancies represented in literature with hypercalcemia, there are only a handful from endometrial carcinoma. Here we describe an atypical case of a patient with endometrial carcinoma who presented with symptomatic hypercalcemia. This case report investigates the atypical presentation of an endometrial carcinoma.

## Introduction

Among etiologies of hypercalcemia of malignancy, the most frequent primary malignancy is the lung (30%), then gastrointestinal (13%), urogenital (15%, of which endometrium was one in 18), hematologic (12%), head and neck (10%), breast (8%), unknown primary (8%), and skin (3%) [1].

Cancer can disrupt the surrounding structures as well as dysregulate metabolic processes in its quest to grow. Hypercalcemia of malignancy has been found to affect 2% to 8.5% of those with malignancies with significant variability depending on the type of malignancy and time of the study [2-4]. Malignancies may disrupt calcium homeostasis through osteolytic processes or mediated through parathyroid hormone-related peptide (PTHrP), 1,25-dihydroxy vitamin D, or parathyroid hormone (PTH) [5].

In this case report, we will elaborate on the presentation, workup, and management of a patient presenting with hypercalcemia of malignancy due to a primary malignancy of endometrial carcinoma. We will also further elaborate on the mechanism involved that may warrant further investigation.

## Case presentation

A female in her late 60s with no known significant past medical history presented to the emergency department with 3-5 days of worsening fatigue and weakness. The patient also complained of associated nausea, vomiting, left lower quadrant abdominal pain, and falls. The exam on admission found some word-finding difficulties as well as difficulty with concentration and recall. She had not been evaluated by a physician in more than 10 years. Her home medications included a multivitamin, calcium, and vitamin D 2000 iu daily.

**Figure 1:**
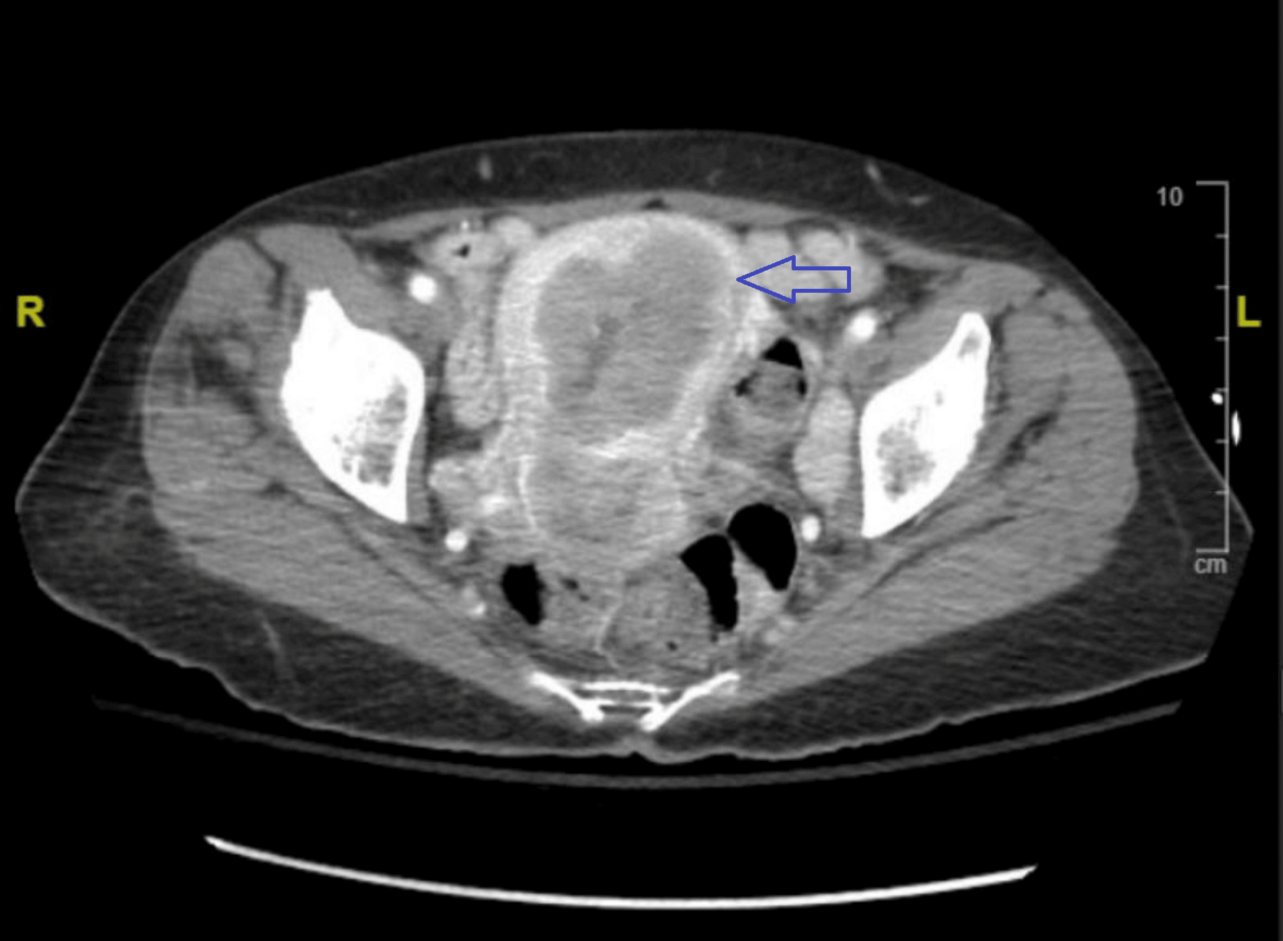
Computed tomography of the abdomen and pelvis demonstrating uterus with endometrial enlargement, the blue arrow points to the uterus

**Figure 2:**
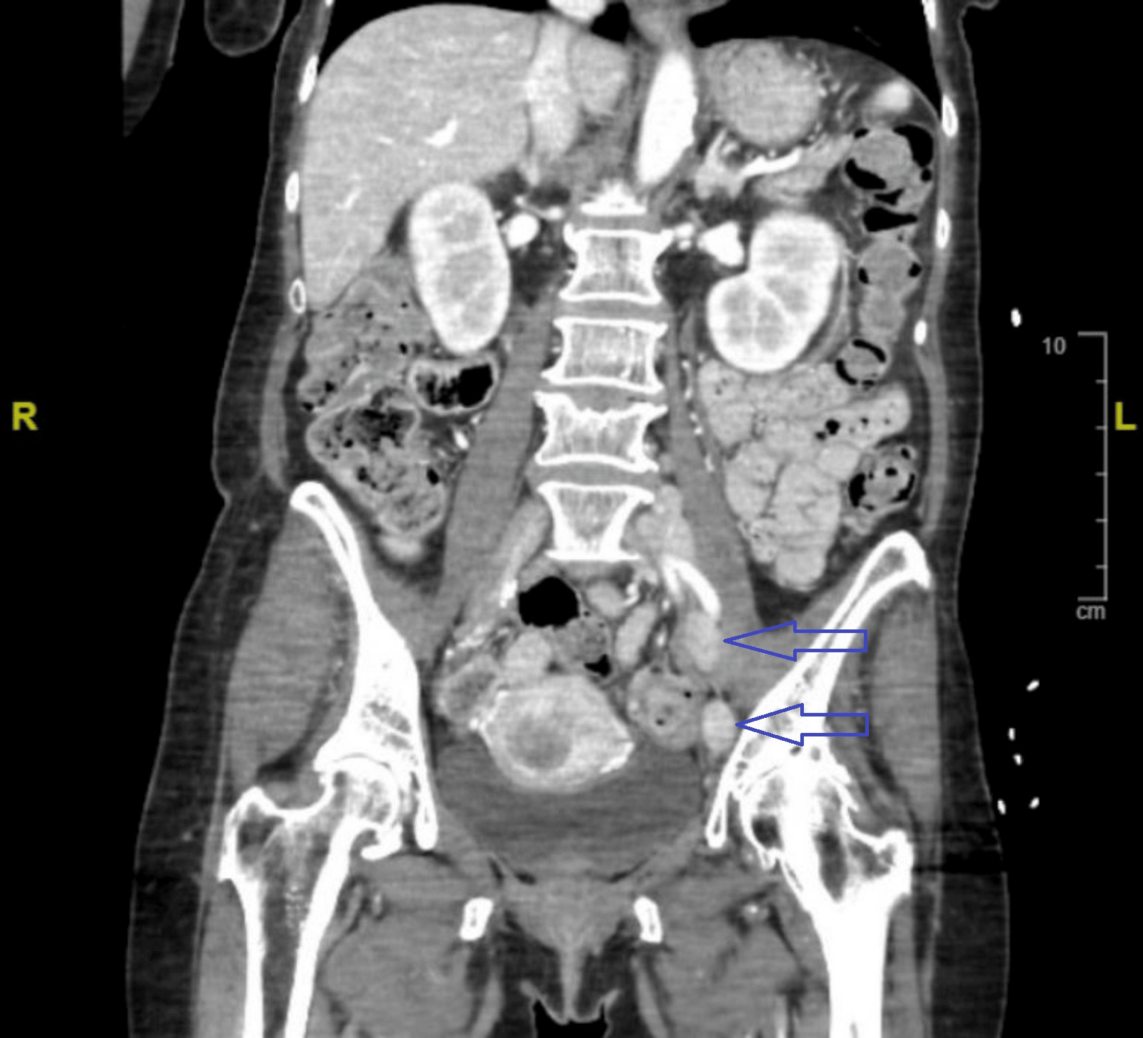
Computed tomography of the abdomen and pelvis demonstrating left inguinal chain lymphadenopathy with blue arrows pointing to the inguinal chain lymphadenopathy

Admission vitals showed the patient was afebrile and hypertensive (blood pressure: 197/102). Labs on admission showed elevated bicarbonate levels and hypercalcemia (Ca: 16.4 mg/dL (ref range: 8.5-10.1)) (Table 1). Additional labs showed serum phosphorus of 2.1 mg/dL (ref range: 2.7-4.5 mg/dL), 25-vit D of 83.0 ng/mL (ref range: 25-100 ng/mL), a normal kappa-lambda (k/l) ratio, and parathyroid hormone resulted at 42.6 pg/mL (ref range: 9.0-73.0 pg/mL). PTHrP was 40 pg/mL (ref range: 10-12 pg/mL). 25-hydroxy vitamin D on admission was 83 ng/mL (ref range: 25-100 ng/mL). CT imaging of the abdomen and pelvis with contrast revealed a nonspecific mass-like enlargement of the uterine endometrium highly concerning for neoplasm as well as large left inguinal chain lymphadenopathy concerning for metastasis. Additionally, in the right adnexal/inguinal chain region, an area of abnormally enhancing 3.3 cm mass-like focus directly adjacent to a loop of small bowel was read as possibly representing adenopathy versus local invasion of tumor (Figures 1, 2).

**Table 1:** Initial presentation labs BUN: blood urea nitrogen; SGOT: serum glutamic oxaloacetic transaminase; SGPT: serum glutamic pyruvic transaminase

Test	Latest reference range	Value
Glucose	70-99 mg/dL	111
BUN	7-18 mg/dL	33
Creatinine	0.55-1.02 mg/dL	1.07
Sodium	136-145 meq/L	135
Potassium	3.6-5.0 meq/L	3.5
Chloride	98-107 meq/L	98
Carbon dioxide	21-32 meq/L	36
Calcium	8.5-10.1 mg/dL	16.4
Bilirubin, total	0.2-1.0 mg/dL	0.4
Alkaline phosphatase	45-117 U/L	85
SGOT (AST)	15-37 U/L	51
SGPT (ALT)	13-56 U/L	37
Protein, total	6.4-8.2 g/dL	7.8
Albumin	3.4-5.0 g/dL	3.0
Anion gap	6-16 meq/L	1
Globulin	2.4-3.5 g/dL	4.8
A/G ratio	1.1-2.2	0.6
Lipase	73-393 U/L	103
Ionized calcium	1.13-1.32 mmol/L	1.33

With initial treatment with fluids, calcitonin, and zoledronic acid, the patient’s serum calcium decreased to 9.4 mg/dL on day 3 of admission. From the initial measured value of 1.33 mmol/L, the ionized calcium decreased to 1.18 mmol/L (normal) with some symptomatic improvement by days 2-3 of admission.

A positron emission tomography (PET) scan revealed metastatic adenopathy involving the left pelvic sidewall, left internal iliac, bilateral common iliac, and retrocaval nodal stations. The patient subsequently underwent a total abdominal hysterectomy with upper vaginectomy and bilateral salpingo-oophorectomy as well as an excisional biopsy of the pre-caval lymph node. Surgical pathology report of the uterus, cervix, and upper vagina showed dedifferentiated endometrial carcinoma with lymphovascular invasion. The surgical pathology of the pre-caval lymph node showed metastatic endometrioid carcinoma (Grade 2-3) with foci of necrosis.

## Discussion

Although hypercalcemia is a fairly common paraneoplastic syndrome, it is rarely attributed to a primary gynecologic malignancy. Hypercalcemia of malignancy has been seen to be associated with a relatively poor prognosis. One single-center retrospective study showed that mortality was 50% within one month of treatment and 75% within three months of treatment [6].

In our case, other possible etiologies of hypercalcemia in terms of endocrine etiology and primary alternative malignancy were ruled out with appropriate testing. We found that with the elevated PTHrP, normal PTH, and normal vitamin D levels, hypercalcemia of malignancy was highly likely. While the workup and treatment of this patient’s hypercalcemia is not entirely unique, it is rare enough to provoke curiosity as to why this is an uncommon occurrence.

A handful of gynecologic malignancies, such as endometrial sarcoma, endometrioid adenocarcinoma, ovarian clear cell adenocarcinoma, and uterine carcinosarcoma, have documented hypercalcemia associated with their disease processes [7-10]. In published literature, even some benign gynecologic pathologies, such as uterine fibroids and uterine leiomyoma, documented to cause hypercalcemia are few and limited [11,12]. A single institution retrospective study looking at patients from The University of Texas M. D. Anderson Cancer Center (Houston, USA) with data from 5620 patients being treated for gynecologic malignancies showed 5% had hypercalcemia and, of those 5%, 35% had endometrioid carcinoma [13].

Since gynecologic malignancies as a source of hypercalcemia are few and far in-between, it may be prudent to also further investigate benign gynecologic pathologies as well as malignancies contributing to hypercalcemia to better understand the biochemical mechanisms driving the hypercalcemia.

Some mechanisms of hypercalcemia of malignancy broken down by frequency are hypercalcemia associated with cancer (20%), osteolytic, humoral hypercalcemia (80%), 1,25(OH)2D-secreting lymphoma (<1%), and ectopic hyperparathyroidism (<1%) [14]. PTHrP is implicated in bone metastasis with breast and colon cancer, and some studies have elucidated estrogen and progestin hormonal regulation involvement with PTHrP [15]. In normal physiology, PTHrP can impact signaling through g-protein-coupled receptors or via nuclear translocation. PTHrP can work as an autocrine or paracrine signal where it can undergo nuclear translocation to likely affect regulation of vascular smooth muscle proliferation. Furthermore, PTHrP has also been seen in studies to be involved in maternal fetal calcium transport, fetal development (in murine studies), endochondral bone formation, and lactational bone loss [16]. In endometrial tissue in particular, PTHrP expression varies chronologically. An in-vitro study of human endometrial tissues demonstrated a significant difference in the PTHrP mRNA expression between the proliferative and secretory phases without difference in PTH/PTHrP receptors in the tissue [17]. Another in vitro study found that placental and uterine decidua parietalis had expression of PTHrP without PTHrP being detected in the maternal or fetal blood [18]. Since PTHrP is evidently closely regulated in gynecologic tissues, it may be prudent to investigate further whether the normal regulatory mechanisms of PTHrP expression in such tissues contribute to the differences in rates of PTHrP-related hypercalcemia in gynecologic malignancies.

Hypercalcemia of malignancy is associated with poor prognosis in patients with malignancy [6]. Further investigating the biomolecular mechanisms driving hypercalcemia in gynecologic malignancies may allow us to better appreciate associated morbidity and targeted treatment options in gynecologic and non-gynecologic malignancies involving dysregulated PTHrP expression and subsequent hypercalcemia.

## Conclusions

Calcium regulation involves various signaling pathways to maintain homeostasis. Depending on the organ system, the expression of receptors and chemical signals affecting calcium movement intracellularly may vary. Disruption of calcium metabolism is often seen in various organ systems, but it is not as commonly seen in primary gynecologic malignancies. We hope this case study brings additional light to a field that warrants further investigations into the biomolecular mechanisms of hypercalcemia of malignancy given the different distribution of etiologies of primary malignancies causing hypercalcemia.
